# Rheological Characterization of Chapatti (Roti) Enriched with Flour or Paste of House Crickets (*Acheta domesticus*)

**DOI:** 10.3390/foods10112750

**Published:** 2021-11-10

**Authors:** Habiba Khatun, Mik Van Der Borght, Mohammad Akhtaruzzaman, Johan Claes

**Affiliations:** 1 Research Group for Insect Production and Processing, Faculty of Engineering Technology, Department of Microbial and Molecular Systems, KU Leuven, Kleinhoefstraat 4, 2440 Geel, Belgium; habiba.khatun@kuleuven.be (H.K.); mik.vanderborght@kuleuven.be (M.V.D.B.); 2 Department of Food Science and Nutrition, Faculty of Engineering, Hajee Mohammad Danesh Science and Technology University, Dinajpur 5200, Bangladesh; 3 Institute of Nutrition and Food Science, University of Dhaka, Dhaka 1000, Bangladesh; makhtar@du.ac.bd

**Keywords:** *Acheta domesticus*, chapatti, wheat flour, rheological properties, textural properties

## Abstract

Addition of edible insects to food products may improve the nutritional status but can also influence their techno-functional properties. This study investigates the impact of supplementing wheat flour by cricket flour or paste at different levels (5–15%) on the rheological and textural properties of flour, dough, and baked chapatti. Addition of freeze-dried cricket flour resulted in the highest water absorption. The storage modulus increased at higher level (10–15%) of supplementation to wheat flour indicating an increased dough consistency. Similarly, biaxial extension of the dough showed an increased resistance to extension and decreased extensibility at higher level of supplementation due to a reduced strength of the gluten network. Uniaxial extension of baked chapatti showed less extensible and harder chapatti with the addition of a higher amount of cricket flour or paste. At lower level (5%), incorporation of cricket flour resulted in chapatti with textural properties comparable to the reference. Oven dried cricket powder is suggested as the best option for incorporating in chapatti dough to improve food security in Asian Countries.

## 1. Introduction

Chapatti, also known as “roti”, is a single-layered flatbread prepared from whole wheat flour which is a typical staple diet in South Asian countries. Freshly prepared chapatti with soft, pliable texture and slightly chewiness is desirable, and serves as a major source of energy and protein for millions of people [1,2]. Being a staple diet, chapatti could act as an effective vehicle to improve a large population’s nutritional status. Although several efforts have been made to improve the nutritional status of chapatti by incorporating different pulses, leafy vegetables and different types of fiber, investigation on the technological functionality of chapatti is scarce [3]. For instance, the addition of different bran types to wheat flour significantly influenced pasting and mixing properties of the flour used in chapatti making [4,5]. The Alveo-consistography results of chapatti dough enriched with spinach powder showed an increase in water absorption and tenacity and a decrease in extensibility of chapatti dough [6]. In another study, Mehfooz et al. (2018) reported that water absorption and storage modulus increased while dough extensibility decreased due to the addition of barley husk at 5–30% which ultimately resulted in increased hardness and reduced extensibility of baked chapatti [7].

Edible insects are rich sources of dietary protein, fat, amino acids, fatty acids and fiber, and can be considered as an interesting alternative to the conventional animal sources [8,9,10,11]. Along with an excellent nutritional profile, pointed out as early as 1975 by Meyer-Rochow [12], generally edible insects are sustainable food sources with a high feed conversion efficiency and relatively less greenhouse gases emission [13,14]. Recently, different findings showed that using insect powder to nutritionally fortify baked products also alters the technological and sensory features of the end product [15,16,17]. For instance, a decreased bread volume when adding cricket (*Acheta domesticus* Linnaeus 1758) or mealworm (*Tenebrio molitor*) powder was observed by Cappelli et al. (2020) [18]. A negative linear correlation between the amount of added insect powder and the dough technological parameters was found by Osimani et al. (2018) and de Oliveira et al. (2017) by incorporating cricket (*A. domesticus*) and cockroach (*Nauphoeta cinerea*) powder, respectively [17,19]. González et al. (2019) reported that breads containing larval *Hermetia illucens* or larval *Tenebrio molitor* flour had lower specific volumes than wheat flour bread, while *A. domesticus* did not affect the specific volume [20]. In contrast, Roncolini et al. (2019) reported an improved specific volume and softer bread when mealworm (*T. molitor*) was added to wheat flour dough at 5–10% [21].

As such, although extensive investigation of bread has been conducted, reports on the rheological properties of chapatti dough and baked chapatti are quite limited. In addition, there are no published data on the incorporation of edible insects into a chapatti. It was reported that flour with higher water absorption capacity resulted in improved textural properties [22]. For this reason, freeze dried cricket flour might be more suited compared to oven dried crickets because of its higher water absorption capacity [16]. However, the operational cost of freeze drying is higher than of oven drying. Therefore, both oven and freeze-dried crickets will be studied. Cricket paste is included because of its easy production procedure. This research investigates the rheological characteristics of flour, dough and baked chapatti enriched with freeze and oven dried powder or blanched paste from house crickets (*A. domesticus*) using small and large deformation mechanical tests.

## 2. Materials and Methods

### 2.1. Raw Materials 

Commercial (All bake) white wheat flour (moisture 12.25%, protein 11.2%, fat 1.2%, ash 0.6%, fiber 3.0%) was purchased from a local market (Geel, Belgium). Adult house crickets (*Acheta domesticus*; Insecta; Orthoptera; Gryllidae) (protein 67–69 g/100 g dry matter, fat ∼15 g/100 g dry matter, ash ∼6 g/100 g dry matter, fiber ∼8 g/100 g dry matter) [10] were purchased from Nusect (Ledegem, Belgium) and starved overnight at 3–4 °C temperature. They were killed by blanching at boiling temperature for 40 s. Immediately after blanching, crickets were placed in chilled water (8 ± 1 °C temperature) for 1–2 min to avoid overcooking. After draining of the water, the crickets were stored at −20 °C in a sealed polythene bag.

### 2.2. Preparation of the Cricket Flour and Paste

To obtain freeze-dried powder, frozen crickets were ground using a kitchen grinder (AR110830, Moulinex, Écully, France) and placed in a freeze drier (Lyovapor L200, Büchi) for 40 h. The obtained powder was further ground (DPA141, Moulinex) and sieved (mesh size < 1.18 mm) to discard coarse particles. To obtain oven-dried powder, thawed crickets were placed in a forced-air oven (UFB500, Memmert, Büchenbach, Germany) at 65 °C for 17 h. Dried crickets were ground (DPA141, Moulinex) to powder and sieved to discard larger fractions. The paste sample was obtained by grinding thawed crickets to a paste using a kitchen grinder (AR110830, Moulinex). Freeze-dried (AFD) and oven-dried (AOD) powders were stored in a sealed polythene bag at 4 °C temperature until further analysis while paste (APT) was prepared instantly before each experiment.

### 2.3. Formulations

Wheat flour and cricket flour blends were formulated by substituting wheat flour at 5%, 10% and 15% level with freeze dried and oven dried cricket flour, coded respectively as AFD 5%, AFD 10%, AFD 15% and AOD 5%, AOD 10% AOD 15%. Cricket paste was used at the same levels (APT 5%, APT 10%, APT 15%). Batches with 100% wheat flour were used as control.

### 2.4. Particle Size Distribution

Particle size distribution of wheat flour and cricket flour was measured by a laser diffraction particle size analyzer (Beckman Coulter Inc., LS 13 320, FL, USA) using the Tornado Dry Powder System. The wavelength of the main illumination source was 750 nm with a minimum obscuration of 4%. The intensity of the scattered laser light was detected and transformed to particle sizes by the Fraunhofer model.

### 2.5. Rheological Properties of Flour

#### 2.5.1. Farinograph Characteristics

Dough mixing characteristics of wheat flour (WF) and wheat flour–cricket flour (CF) blends were analyzed in a Farinograph (constant flour weight procedure) with a 50-g mixer bowl (Brabender, Duisburg, Germany) according to the AACC International method 54-21.02. The parameters determined were water absorption (WA) to reach a dough consistency of 500 BU, dough development time and stability.

#### 2.5.2. Pasting Properties

Pasting properties of wheat flour and cricket flour blends were analyzed using a rheometer (MCR-301, Anton Paar, Ostfildern, Germany) equipped with a starch cell (C-ETD 160) and stirrer probe (ST 24-2D/2V/2V-30). The flour suspension was prepared by dissolving 1.8 g of flour (wheat flour or cricket flour blends) in 15.0 mL water. Suspensions were first mixed for 10 s and held at 50 °C for 50 s then heated from 50 to 95 °C at 0.1 °C/s, held at 95 °C for 5.0 min, after which the paste was cooled from 95 to 50 °C, and held at 50 °C for 2 min. Parameters obtained were peak viscosity, breakdown viscosity, setback viscosity and pasting temperature.

### 2.6. Rheological Properties of Dough

#### 2.6.1. Dough Preparation

To obtain a dough with good handling properties 52.4 g tap water and 2.0 g table salt (NaCl) were added to 100.0 g flour (either WF or CF blends) and mixed to dough by a kneading machine (Kenwood Major Titanium KMM020, New Lane, UK) using a dough hook for 5.0 min at speed 3. From each batch, the dynamic properties and biaxial extensibility of the dough and the texture measurement of baked chapatti were determined. To prepare the dough with cricket paste, the moisture content of the paste was used to calculate the water addition. However, the reference amount of 52.4 g water was insufficient to obtain a homogeneous dough probably because of the inclusion of water in the paste structure. Therefore, for the three paste samples 62.4 g water was used.

#### 2.6.2. Dynamic Oscillatory Measurements

Dynamic rheology of the dough sample was measured by a rheometer (model MCR 301, Anton Paar, Ostfildern, Germany) using parallel serrated plates (25 mm diameter) with a strain sweep from 0.01 to 100% at a frequency of 1 Hz. After resting for 12 min an aliquot of dough was taken from the center and placed between the plates conditioned previously at 25 °C with 1.5 mm gap. Excess sample was trimmed, and the edge of the sample was covered with silicone oil to avoid sample dehydration during the test. The test started after a resting time of 2 min between the plates. Each formulation was prepared three times and each preparation was analyzed once.

#### 2.6.3. Dough Extensibility

Biaxial extensibility measurement of the dough was carried out using a texture analyzer (Stable Micro system TA.XTplus, Godalming, Surrey, England) equipped with a tortilla/pastry rig (HDP/TPB) on a heavy duty platform with a 5 kg load cell. After resting for 5 min, the dough was sheeted to approx. 1.9 mm thickness by a pasta sheeter (Model KAX 980 ME, Kenwood, Treviso, Italy) and cut into a specific dimension of 10 cm × 10 cm. The ball probe (SMSP/1 SP) started at 80 mm above the platform and travelled at a test speed and post-test speed of 1.0 and 10.0 mm/s, respectively over a distance of 75 mm. Data acquisition rate was 200 pps. Three samples from separate batches were evaluated and the results are presented as the maximum force or tenacity, the force to extend 30 mm and extensibility at maximum force.

### 2.7. Chapatti Making

Chapatti was prepared according to the method described by Thakur et al. (2017) with slight modification [23]. After resting for 30 min the dough was sheeted to approx. 1.9 mm thickness by a pasta sheeter (Model KAX 980 ME, Kenwood, Treviso, Italy) and cut into a dimension of 12 cm × 10 cm. Immediately after sheeting, chapatti was baked at 215 ± 2 °C in a tortilla maker (Princess, Tilburg, The Netherlands). One side of the chapatti was baked for 90 s and then flipped to bake the other side also for 90 s. After baking it was cooled to room temperature (25 ± 2 °C) for 7 min and tested for uniaxial extension.

#### Chapatti Texture

The texture of the baked chapatti was evaluated using a texture analyzer (TA.XTplus, Stable Micro system, Godalming, Surrey, England) fitted with a tensile grip (A/TG). Rectangular strips of 7.0 cm × 3.5 cm dimension were cut out near the central portion of chapatti. The cut strip was analyzed by placing it between two clamps of the texturometer at a distance of 22 mm. The chapatti strip was pulled apart until it ruptured by using a load cell of 5 kg at a travelling speed of 1 mm/sec. The extensibility test was conducted using the return to start option. The force to extend the chapatti strip 1 mm, force to rupture, and the distance of extension were recorded. The mean of six measurements from three batches was calculated.

### 2.8. Statistical Analysis

One-way ANOVA was used to check the impact of adding cricket flour and paste on all the rheological and textural properties using SPSS (Version 23, IBM Corporation, Armonk, NY, USA) while normality was tested by Shapiro–Wilk test and homogeneity was tested by Levene’s test. Tukey’s post-hoc test was used to separate the means at 5% level of significance. Kruskal Wallis test with Benferroni post-hoc test was used for unequal and non-homogenous samples.

## 3. Results and Discussion

### 3.1. Particle Size Distribution

Particle size of flour is important as it influences the dough rheology and the texture of final products due to the variations of constituents in different fractions of particles and the different water binding capacity as function of particle size [24]. Wheat flour had the smallest particle size, while freeze and oven cricket flour only had a slightly different size, of which the latter had the largest size (Table 1). The variation in particle size was expected as freeze-dried cricket powder was subjected to grinding twice while oven dried cricket was subjected to one grinding step during the preparation.

### 3.2. Rheological Properties of the Flour

#### 3.2.1. Effect of Cricket Flour on Dough Mixing Properties

Incorporation of oven-dried, and freeze-dried cricket powder showed significant effects on the dough mixing properties as measured by the farinograph (Table 2). Generally, these effects were more pronounced with a higher substitution level. Water absorption increased with the increase of cricket powder in wheat flour dough from 51.30% to 55.10%. This increase of water absorption can be explained by the fact that cricket powder contains a four times higher protein and two times higher fiber content than wheat flour. The increase of Farinograph water absorption due to the higher cricket flour content has also been reported by Osimani et al., 2018 [17] and Cappelli et al., 2020 [18] when partially substituting wheat flour by cricket flour (*A. domesticus*).

Substitution of freeze-dried cricket flour (AFD) had a higher effect on water absorption than that of oven dried cricket flour (AOD) resulting in the highest water absorption in the formulation AFD 15%. Lucas-González et al., (2019) also reported the highest water absorption for freeze dried cricket (*A. domesticus*) [16]. Higher water absorption by AFD might be due to its more porous structure compared to oven dried samples [25]. The smaller particle size of AFD (Table 1) could also cause a higher water absorption [26].

Cricket flour addition also increased the dough development time significantly (*p* < 0.05) ranging from 1.83 min for wheat flour to 5.27 min for AOD 15%. Similar effects on dough development time due to addition of fiber and protein rich ingredients were reported by several authors [27,28,29]. The increase of dough development time could also be explained by the interaction of gluten and cricket protein leading to a delay in hydration and development of the gluten network [29]. Dough stability decreased significantly compared with the control owing to the increase of the amount of cricket powder. Dough stability represents a higher tolerance to mixing, for which gluten develops a three-dimensional viscoelastic structure [30]. The addition of cricket powder could interrupt the development of the gluten network structure by diluting the gluten concentration resulting in lower dough stability. In contrast, Osimani et al. (2018) reported that the farinograph properties of wheat flour did not change up to 10% supplementation with *A. domesticus* powder, but further addition had a significant effect [17]. This different result might be due to the lower protein (53.51 g/100 g DM) content of the *A. domesticus* flour used by the author than that of our study (67 g/100 g DM)**.** This is supported by Cappelli et al. (2020) who showed a significant positive relationship between water absorption and protein content of *A. domesticus* [18].

#### 3.2.2. Effect of Cricket Flour on Pasting Properties of Wheat Flour and Cricket Flour Blends

The pasting properties are important as these are used in predicting the gelatinization behavior and pasting ability of flour. Kundu et al. (2017) showed that peak viscosity and breakdown viscosity had a significantly positive correlation with chapatti extensibility [31].

**Figure 1:**
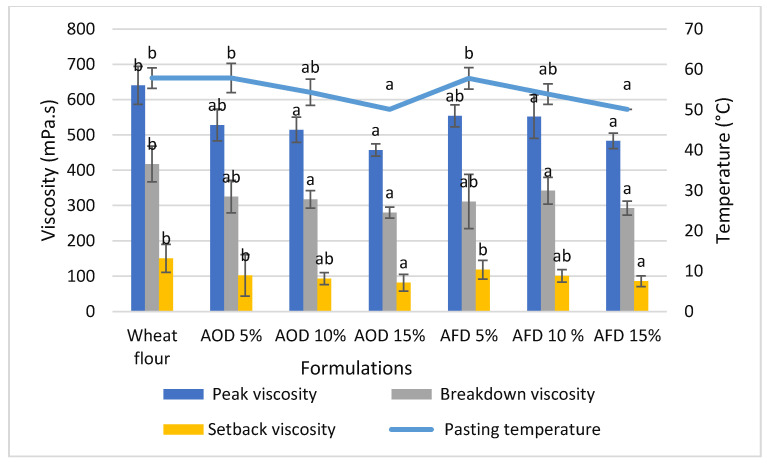
Effect of cricket flour on pasting properties of wheat flour and cricket flour blends. Results are presented as the mean of three replicates. Error bars correspond with ± standard deviations. Different letters mean statistically different values (*p* < 0.05). WF = Wheat Flour; AOD = *A. domesticus* oven dried; AFD = *A. domesticus* freeze dried.

Peak viscosity decreased with a higher substitution level (Figure 1). For 10% and 15% substitution, this reduction was also significant (*p* < 0.05). Similarly, the breakdown viscosity and setback viscosity decreased with an increasing cricket powder substitution. These lower viscosities indicate less pronounced variations in the viscosity profile during pasting. The lower setback viscosity due to addition of cricket flour indicates a lower gel stability and lower sensitivity to retrogradation [32], probably caused by the more difficult re-association of the starch molecules due to the higher protein and lipid content.

The result is also in agreement with the findings reported by several authors when supplementing wheat flour with wheat bran as a source of insoluble fiber [4], chickpea flour as a source of protein [27], different fats and oils [33]. The pasting temperature did not change significantly up to 10% substitution of wheat flour while it decreased with the addition of 15% cricket flour. The decreasing trend of the pasting temperature was also reported by Indriani et al. (2020) when studying the addition (20%) of Bombay locust (*Patanga succincta*) flour to rice flour [34]. Khan et al. (2015) also reported a lower pasting temperature when spinach powder containing high fiber was added to wheat flour [6]. The increased number of proteins, lipids and fibers dilute the starch content of the flour and restrict starch swelling and gelatinization during cooking, thereby lowering the peak viscosity [35,36]. The decrease of the pasting temperature may be attributed to the lower starch proportion in the system, but also to the water release that is absorbed by proteins and fiber during heating [37].

### 3.3. Rheological Properties of Dough

#### 3.3.1. Effects of Cricket Flour on Dynamic Moduli

A dynamic oscillatory test was conducted to study the viscoelastic properties of the dough prepared from different blends of wheat flour and cricket paste along with freeze-dried and oven-dried cricket powder. To facilitate direct comparisons between the effects of adding cricket flour to wheat flour dough, a constant water amount was added. However, more water was required to make a homogenous dough with cricket paste (as described in Section 2.6.1).

**Figure 2:**
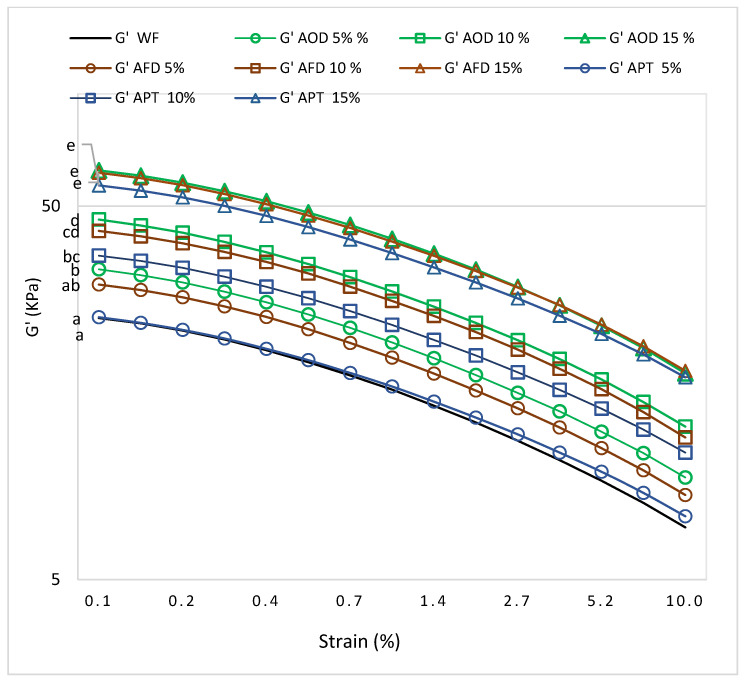
Elastic module (G’) as a function of strain obtained in dynamic oscillatory test. Results are presented as mean of three replicates. Different letters indicate statistically different values (*p* < 0.05). WF = Wheat flour; AOD = *A. domesticus* oven dried; AFD = *A. domesticus* freeze dried; APT = *A. domesticus* paste.

The mechanical spectra produced by the strain sweeps always showed a higher storage modulus (G’) compared to the loss modulus (G’’, results not shown), which indicate a viscoelastic solid behaviour of all the doughs. As can be seen in Figure 2, a higher substitution of wheat flour by cricket flour resulted in increasing values for G’ indicating an increased dough stiffness. At 0.10% strain, the increasing effect by AOD and AFD did not differ significantly, but the values for AOD were for all substitution levels higher than for AFD. It was, however, expected that AFD would give higher G’ values due to the higher water absorption (Section 3.2.1) compared to AOD. Maybe the porous structure of freeze-dried cricket flour exerted lower resistance towards the applied shear stresses but this requires further and more detailed research.

The same increasing trend with increasing substitution is present for paste, but due to the higher water addition, these values cannot be compared with the results for cricket flour.

Several factors can contribute to the changes of storage module and loss module of the formulated dough. Firstly, the lower water content of cricket powder compared with wheat flour might contribute to the higher value of G’, as both the moduli are sensitive to water that acts as a plasticizer in the dough system [38]. Secondly, a higher fiber content could exaggerate the increasing effect on G’ and G” by absorbing more water and leaving less available water for starch and gluten. Thus, the limiting effect of water plasticization led to an overall increase of the mechanical characteristics of the dough [39]. Chitin content was also reported to increase G’ and G’’ value by interacting with macromolecules through electrostatic interaction and hydrogen bonding [40,41]. Thirdly, the higher protein content of the cricket flour could increase the dough consistency by creating a robust crosslinked structure in doughs resulting in higher G’ and G” [42]. Increased viscoelastic moduli due to Bombay locust (*Patanga succincta L*.) powder and cricket (*A. domesticus*) protein hydrolysates addition to rice flour and corn masa dough were also reported by Indriani et al. (2020) and Luna et al. (2021), respectively [34,43].

#### 3.3.2. Effects of Cricket Flour on Biaxial Extensibility

Dough resistance to deformation or tenacity could be used as a predictor of gas retention capacity of the dough during baking, while extensibility of dough is used as an indicator of the handling characteristics of the dough.

**Figure 3:**
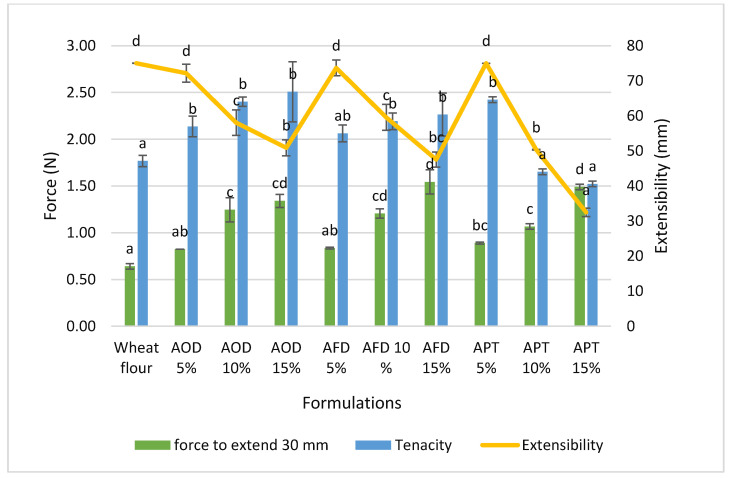
Effect of cricket flour on biaxial extensibility of wheat flour and cricket flour dough. Results are presented as mean of three replicates. Error bars correspond with ± standard deviations. Different letters mean statistically different values (*p* < 0.05). AOD = *A. domesticus* oven dried; AFD = *A. domesticus* Freeze dried; APT = *A. domesticus* paste.

The biaxial extensibility tests showed that both the tenacity and extensibility of the dough were significantly affected with the addition of cricket powder as shown in Figure 3. With an increased substitution level of cricket powder, the tenacity ranged from 1.77 N for WF to 2.51 N reflecting the increased dough hardness, which was also observed in the oscillatory tests (Figure 2). All the formulated doughs ruptured at the maximum force when the substitution increased.

Dough extensibility is another parameter used to predict baked chapatti as dough with a higher extensibility gives better chapatti with better texture and mouth feel [43]. Dough extensibility showed a decreasing trend at higher substitution levels. The dough containing paste showed a more pronounced decrease of the extensibility (with only 32.48 mm extension for APT 15%). This small extensibility did not allow the use of force at rupture as a measure for dough strength, as is indicated by the decreasing trend in rupture force with a higher substitution degree. Therefore, the force required to extend the dough to 30 mm was used as a better characteristic value to compare the strength of different doughs (Figure 3). For all substitutions (AOD, AFD and APT), a higher substitution resulted in a significantly higher force and thus a stronger dough, which was also observed when manipulating the dough. It should be noted that a low extensibility is also bad in connection with handling abilities.

Tenacity and extensibility of dough depends on gluten strength which could be disrupted by protein and fiber present in cricket powder resulting in a weak gluten network that could easily break down during textural analysis. Cappelli et al. (2020) reported that higher level (10% and 15%) supplementation of wheat flour with mealworm (*T. molitor*) and cricket (*A. domesticus*) powder resulted in an increased tenacity and decreased extensibility of the bread dough [18]. Although the interaction between fiber and gluten cannot be generalized due to the diverse nature and chemical structure of fiber composition, many researchers showed that due to the competitive water absorption the type of fiber can impart partial dehydration of gluten leading to a conformational change in the gluten matrix and a collapse of the polymeric gluten network [44]. A similar decrease in the extensibility of wheat flour chapatti dough substituted by spinach powder and barley husk was also found by Khan et al. (2015) and Mehfooz et al. (2018) [6,7].

### 3.4. Effects of Cricket Flour on Uniaxial Extensibility of Baked Chapatti

**Figure 4:**
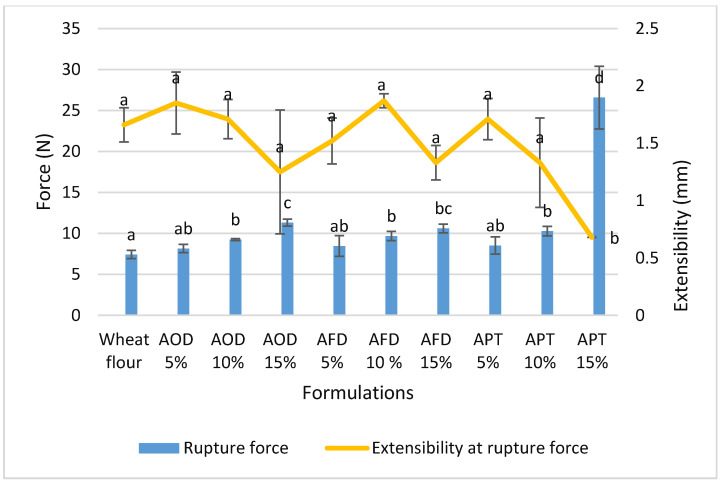
Uniaxial extensibility of baked chapatti. Results are presented as mean of six replicates. Error bars correspond with ± standard deviations. Different letters mean statistically different values (*p* < 0.05). AOD = *A. domesticus* oven dried; AFD = *A. domesticus* freeze dried; APT = *A. domesticus* paste.

Excellent quality chapattis are fully puffed chapatti with optimum chewiness and lower tearing resistance as the result of a higher number of gliadins and fewer glutenins [45]. The textural characteristics of baked chapattis are presented in Figure 4. Chapatti without cricket powder required a lower tearing force, while the addition of cricket powder to the dough increased the force by up to 300%. However, at a low substitution of 5%, there was no significant increase of the rupture force. Despite the higher water absorption of freeze-dried cricket powder compared to oven dried cricket flour, the baked chapatti did not show different textural properties between these two powder types. On the other hand, optimizing the handling properties of dough containing cricket paste was troublesome, which resulted also in a worse chapatti texture at higher level of addition.

The uniaxial extensibility parameters of baked chapatti did not significantly differ between the two types of cricket flour. The extensibility at rupture force showed a non-significant decreasing trend (Figure 4) indicating similar elasticity compared to wheat flour chapatti. Only with 15% cricket paste was the extensibility significantly lower (combined with a very high rupture force). It can be seen from the result that, although 5% paste inclusion resulted in softer dough compared to AFD and AOD (Figure 2), the baked chapatti did not differ significantly with 5% AOD and AFD.

These results suggest that addition of cricket powder or paste interferes with the formation of an extensible gluten network resulting in a harder chapatti. A similar trend of increasing hardness in bread enriched with higher (10–20%) crickets and cockroach flour was reported by several authors [19,20,46]. The water binding capacity of fiber could also negatively interfere with the development of the starch–gluten structure which led to an increased hardness and a reduced elasticity [47]. Khan et al., (2015), Mehfooz et al., (2018) and Yadav et al., (2010) also reported increased hardness in chapatti with an increased fiber content in the wheat flour [4,6,7].

## 4. Conclusions

Partial substitution of wheat flour with cricket flour improves the nutritional value of bakery products but can also have an impact on the functional properties. The incorporation of cricket flour in chapatti dough altered the Farinograph characteristics and the differences were more pronounced at the higher level of substitution. Freeze dried cricket flour had a more pronounced effect on the mixing properties compared with oven dried flour, with a higher water absorption, longer dough development time and lower dough stability. Unlike mixing properties, pasting properties did not differ between the types of cricket flour but the addition of cricket flour reduced all the pasting viscosities. The storage modulus increased sharply with a higher substitution of cricket flour and paste showing stiffer dough compared to wheat flour dough. This was also confirmed in the biaxial extensibility test of the dough, with a higher tenacity and reduced extensibility, especially at higher levels of substitution (10–15%). The textural properties of the baked chapatti did not differ significantly from the wheat flour reference at 5% supplementation, but higher addition (10–15%) of cricket flour and paste resulted in harder and less extensible chapatti. An addition of a smaller amount (5%) of cricket flour or paste is possible without significantly altering the texture of wheat flour chapatti. Higher amounts of crickets can only be used in combination with hydrocolloids, which will be part of further research, together with a detailed study of the consumer acceptability of the enriched chapatti by sensory analysis.

## Figures and Tables

**Table 1:** Particle size distribution of wheat flour, oven-dried and freeze-dried flour of *A. domesticus*.

Samples	Particle Size µm < Volume *		
	10D	50D	90D
Wheat flour	16.00 ± 0.30	74.74 ± 0.09	156.03 ± 0.29
AOD	101.77 ± 1.44	424.06 ± 7.78	1050.58 ± 82.16
AFD	65.58 ± 0.49	344.04 ± 0.15	855.58 ± 0.15
APT		Nd **	

AOD = *A. domesticus* oven dried; AFD = *A. domesticus* Freeze dried; APT = *A. domesticus* paste. * Particle sizes are presented as mean ± standard deviation, of two replicates. 10D, 50D and 90D represent a diameter where 10%, 50% or 90% of the particles had a smaller particle size. ** Nd = Not determined.

**Table 2:** Mixing characteristics of wheat flour enriched with cricket flour.

Samples	Water Absorption (%)	Dough Development Time (min)	Dough Stability (min)
Wheat flour	51.23 ± 0.12 ^a^	1.83 ± 0.15 ^a^	8.83 ± 0.15 ^c^
AOD 5%	51.30 ± 0.00 ^a^	1.90 ± 0.26 ^a^	9.17 ± 0.42 ^c^
AOD 10%	52.30 ± 0.00 ^b^	5.20 ± 0.20 ^bc^	6.73 ± 0.31 ^b^
AOD 15%	53.60 ± 0.00 ^c^	5.27 ± 0.46 ^bc^	4.00 ± 0.95 ^a^
AFD 5%	52.46 ± 0.81 ^b^	3.00 ± 0.70 ^ab^	6.37 ± 0.57 ^b^
AFD 10%	53.40 ± 0.00 ^c^	4.23 ± 0.40 ^b^	5.77 ± 0.12 ^b^
AFD 15%	55.10 ± 0.00 ^d^	4.57 ± 0.55 ^b^	5.40 ± 0.26 ^b^

Results are presented as mean ± standard deviation, of three replicates. Different letters in a column mean statistically different values (*p* < 0.05). AOD = *A. domesticus* oven dried; AFD = *A. domesticus* freeze dried.

## Data Availability

The data presented in this study is fully contained in the article.
